# Supplementary figures and images for: Postoperative analgesic effects of paravertebral block versus erector spinae plane block for thoracic and breast surgery: A meta-analysis
Source: PLoS One. 2021 Aug 25;16(8):e0256611. doi: 10.1371/journal.pone.0256611 (PMC8386864; doi:10.1371/journal.pone.0256611)

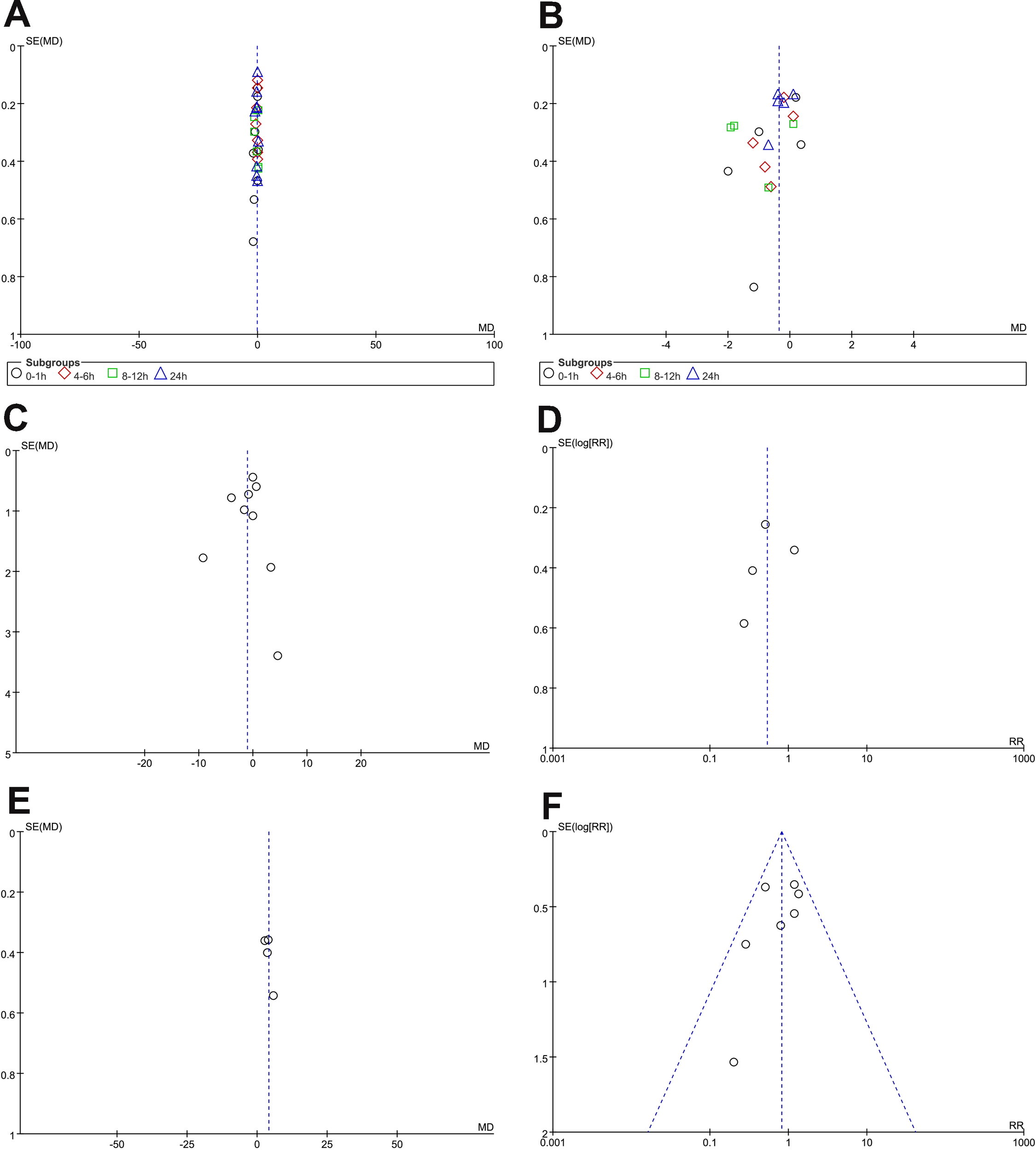

Supplement: S1 Fig — Panel A presents the funnel plot for pain scores at rest at 0–1, 4–6, 8–12 and 24 hours, panel B for pain scores at movement at 0–1, 4–6, 8–12 and 24 hours, panel C for opioid consumption at 24 hours after surgery, panel D for incidence of additional analgesia in 24 hours post-operation, panel E for time required for completing block procedure and panel F for incidence of PONV. (TIF) [file pone.0256611.s002.tif]
