# Supplementary material for: Postoperative analgesic effects of paravertebral block versus erector spinae plane block for thoracic and breast surgery: A meta-analysis
Source: PLoS One. 2021 Aug 25;16(8):e0256611. doi: 10.1371/journal.pone.0256611 (PMC8386864; doi:10.1371/journal.pone.0256611)
Supplement: S1 Table — (DOC) [file pone.0256611.s003.doc]

**S1 Table** Publication bias for each outcome.

|  | Pain scores at rest | | | |  | Pain scores at movement | | | |  | Opioid consumption at 24 hours after surgery | Incidence of additional analgesia in 24 hours post-operation | Time required for completing puncture | Incidence of PONV |
| --- | --- | --- | --- | --- | --- | --- | --- | --- | --- | --- | --- | --- | --- | --- |
|  | 0-1 hours | 4-6 hours | 8-12 hours | 24 hours |  | 0-1 hours | 4-6 hours | 8-12 hours | 24 hours |  |  |  |  |  |
| Egger’s test(P) | 0.118 | 0.644 | 0.961 | 0.240 |  | 0.263 | 0.259 | 0.931 | 0.101 |  | 0.596 | 0.958 | 0.113 | 0.277 |
