# Supplementary material for: Postoperative analgesic effects of paravertebral block versus erector spinae plane block for thoracic and breast surgery: A meta-analysis
Source: PLoS One. 2021 Aug 25;16(8):e0256611. doi: 10.1371/journal.pone.0256611 (PMC8386864; doi:10.1371/journal.pone.0256611)
Supplement: S1 File — (PDF) [file pone.0256611.s004.pdf]

National Institute for Health Research

UNIVERSITY of York
Centre for Reviews and Dissemination

Systematic review

1. * Review title.

Give the title of the review in English

Comparison of paravertebral block versus erector spinae plane block for postoperative analgesia in thoracic surgery and breast surgery: A systematic review and meta-analysis

2. Original language title.

For reviews in languages other than English, give the title in the original language. This will be displayed with the English language title.

3. * Anticipated or actual start date.

Give the date the systematic review started or is expected to start.

15/04/2021

4. * Anticipated completion date.

Give the date by which the review is expected to be completed.

31/05/2021

5. * Stage of review at time of this submission.

Tick the boxes to show which review tasks have been started and which have been completed. Update this field each time any amendments are made to a published record.

Reviews that have started data extraction (at the time of initial submission) are not eligible for inclusion in PROSPERO. If there is later evidence that incorrect status and/or completion date has been supplied, the published PROSPERO record will be marked as retracted.

This field uses answers to initial screening questions. It cannot be edited until after registration.

The review has not yet started: No

Review stage	Started	Completed
Preliminary searches	Yes	No
Piloting of the study selection process	No	No
Formal screening of search results against eligibility criteria	No	No
Data extraction	No	No
Risk of bias (quality) assessment	No	No
Data analysis	No	No

Provide any other relevant information about the stage of the review here.

6. * Named contact.

The named contact is the guarantor for the accuracy of the information in the register record. This may be any member of the review team.

Chang Xiong

Email salutation (e.g. "Dr Smith" or "Joanne") for correspondence:

Dr Xiong

7. * Named contact email.

Give the electronic email address of the named contact.

xiongchang811@163.com

8. Named contact address

Give the full institutional/organisational postal address for the named contact.

Affiliated Jinhua Hospital, Zhejiang University School of Medicine (JinHua Municipal Central Hospital), 365 Renmin East Road, Jinhua, Zhejiang, China

9. Named contact phone number.

Give the telephone number for the named contact, including international dialling code.

17888299176

10. * Organisational affiliation of the review.

Full title of the organisational affiliations for this review and website address if available. This field may be completed as 'None' if the review is not affiliated to any organisation.

Department of Anesthesiology, Affiliated Jinhua Hospital, Zhejiang University School of Medicine (JinHua Municipal Central Hospital)

Organisation web address:

http://www.jhzxyy.cn/

11. * Review team members and their organisational affiliations.

Give the personal details and the organisational affiliations of each member of the review team. Affiliation refers to groups or organisations to which review team members belong. **NOTE: email and country now MUST be entered for each person, unless you are amending a published record.**

Dr Chang Xiong. Department of Anesthesiology, Affiliated Jinhua Hospital, Zhejiang University School of Medicine (JinHua Municipal Central Hospital)
Dr Cheng-peng Han.
Dr Zhi-jian Lan. Department of Anesthesiology, Affiliated Jinhua Hospital, Zhejiang University School of Medicine (JinHua Municipal Central Hospital)

12. * Funding sources/sponsors.

Details of the individuals, organizations, groups, companies or other legal entities who have funded or sponsored the review.

This project is funded by Jinhua Municipal Science and Technology Bureau

Grant number(s)

State the funder, grant or award number and the date of award

Science and technology research program of Jinhua city(No: 2021-4-004)

13. * Conflicts of interest.

List actual or perceived conflicts of interest (financial or academic).

None

14. Collaborators.

Give the name and affiliation of any individuals or organisations who are working on the review but who are not listed as review team members. **NOTE: email and country must be completed for each person, unless you are amending a published record.**

15. * Review question.

State the review question(s) clearly and precisely. It may be appropriate to break very broad questions down into a series of related more specific questions. Questions may be framed or refined using PI(E)COS or similar where relevant.

This systematic review and meta-analysis will compare the analgesic efficacy of paravertebral block (PVB) with erector spinae plane block (ESPB) in adult thoracic surgery and breast surgery.

16. * Searches.

State the sources that will be searched (e.g. Medline). Give the search dates, and any restrictions (e.g. language or publication date). Do NOT enter the full search strategy (it may be provided as a link or attachment below.)

Two of the reviewers (Chang Xiong and Cheng-peng Han) independently sought and retrieved relevant
Studies from electronic databases including PubMed, MEDLINE, EMBASE, Web of Science, Cochrane Central Register of Controlled Trials and Cochrane Library. We also searched for grey literature from other internet resources and retrieved for any relevant references that may have been missed during the literature search. We will not impose any language restrictions.

17. URL to search strategy.

Upload a file with your search strategy, or an example of a search strategy for a specific database, (including the keywords) in pdf or word format. In doing so you are consenting to the file being made publicly accessible. Or provide a URL or link to the strategy. Do NOT provide links to your search **results**.

Alternatively, upload your search strategy to CRD in pdf format. Please note that by doing so you are consenting to the file being made publicly accessible.

Do not make this file publicly available until the review is complete

18. * Condition or domain being studied.

Give a short description of the disease, condition or healthcare domain being studied in your systematic review.

No matter thoracic surgery or breast surgery, postoperative analgesia has always been the focus of attention of anesthesiologists. Postoperative pain without good treatment may cause complications such as unhealing wound, respiratory inhibition, hemodynamic disorder, anxiety and fidgety, leading to prolonged stay of hospital and difficulty of recovery of patients. PVB and ESPB have been popular methods of postoperative analgesia in the recent years because of the popular use of ultrasound. More and more published studies compared PVB with ESPB in terms of the VAS and complications. But these articles were not well-integrated and the results of them have been found contradictory and unconvincing. For example, some of these studies thought the analgesic effect of PVB is similar with ESPB but the other did not think so. We will conduct a systematic review and meta-analysis to assess the efficacy and safety of PVB and ESPB.

19. * Participants/population.

Specify the participants or populations being studied in the review. The preferred format includes details of both inclusion and exclusion criteria.

Patients undergoing thoracotomy surgery or thoracoscopic surgery or breast surgery.

20. * Intervention(s), exposure(s).

Give full and clear descriptions or definitions of the interventions or the exposures to be reviewed. The preferred format includes details of both inclusion and exclusion criteria.

Patients undergoing thoracotomy surgery or thoracoscopic surgery or breast surgery with PVB.

21. * Comparator(s)/control.

Where relevant, give details of the alternatives against which the intervention/exposure will be compared (e.g. another intervention or a non-exposed control group). The preferred format includes details of both inclusion and exclusion criteria.

Patients undergoing thoracotomy surgery or thoracoscopic surgery or breast surgery with ESPB.

22. * Types of study to be included.

Give details of the study designs (e.g. RCT) that are eligible for inclusion in the review. The preferred format includes both inclusion and exclusion criteria. If there are no restrictions on the types of study, this should be stated.

Randomized control trials only

23. Context.

Give summary details of the setting or other relevant characteristics, which help define the inclusion or exclusion criteria.

24. * Main outcome(s).

Give the pre-specified main (most important) outcomes of the review, including details of how the outcome is defined and measured and when these measurement are made, if these are part of the review inclusion criteria.

We designated postoperative pain scores (visual analogue scale, VAS: 0 = no pain, 10 = worst pain
imaginable) during rest and dynamic at postoperatively 2h, 4h, 6h, 8h, 12h and 24h, as the primary outcome.

Measures of effect

Please specify the effect measure(s) for you main outcome(s) e.g. relative risks, odds ratios, risk difference, and/or 'number needed to treat.

Postoperative pain assessment was done using the VAS and the Numerical Rating Scale (NRS). Postoperative pain
severity reported as NRS scores was converted to VAS scores.

25. * Additional outcome(s).

List the pre-specified additional outcomes of the review, with a similar level of detail to that required for main outcomes. Where there are no additional outcomes please state ‘None’ or ‘Not applicable’ as appropriate to the review

Secondary outcomes included: opioid consumption, additional analgesia, postoperative nausea and vomiting (PONV) 24 hours post-operation, and the time required for completing puncture.

Measures of effect

Please specify the effect measure(s) for you additional outcome(s) e.g. relative risks, odds ratios, risk difference, and/or 'number needed to treat.

26. * Data extraction (selection and coding).

Describe how studies will be selected for inclusion. State what data will be extracted or obtained. State how this will be done and recorded.

Relevant data, including author, year of publication, country, number of patients in each group, location of nerve block, local anesthetic dose, surgical approach, duration of surgery and outcomes were extracted independently from eligible articles by two researchers (Chang Xiong and Chengpeng Han). Attempts were made to retrieve raw data for continuous variables from the eligible articles if variables in the full texts were presented as median and range; however, if data could not be extracted, then, the median and range were transformed to the mean ± standard deviation (SD). WebPlotDigitizer was used to extract numerical data if data values were given in a graphical format. Any disagreements arising from the entire process were arbitrated by a third experienced researcher (Zhijian Lan). The primary outcome was postoperative pain scores. The secondary outcomes included opioid consumption, additional analgesia, postoperative nausea and vomiting (PONV) at 24 hours post-operation, and the time required for completing puncture.

27. * Risk of bias (quality) assessment.

State which characteristics of the studies will be assessed and/or any formal risk of bias/quality assessment tools that will be used.

Cochrane risk-of-bias tool for randomized trials will be used for assessment, and conducted independently by two authors, disagreements will be settled by a third author.

28. * Strategy for data synthesis.

Describe the methods you plan to use to synthesise data. This **must not be generic text** but should be **specific to your review** and describe how the proposed approach will be applied to your data. If meta-analysis is planned, describe the models to be used, methods to explore statistical heterogeneity, and software package to be used.

Continuous data on outcomes, including postoperative pain scores, opioid consumption at 24 hours post-operation and the time required for completing puncture were presented as mean difference (MD) at 95% confidence interval (CI). The dichotomous data on outcomes, such as incidence of additional analgesia in 24 postoperative hours and PONV were expressed as the relative risk (RR) at 95% CI. The ?2 test and I^2 statistic were employed to estimate statistical heterogeneity across studies. The I^2 statistic was stratified into three levels: low-level (0–49%), moderate-level (50%–74%), and high-level (75%). The fixed-effects model was used in the event of low-level heterogeneity; otherwise, a random-effects model was applied. For moderate-level and high-level of heterogeneity (I^2 50%), sensitivity analysis or subgroup analysis was performed. Sensitivity analysis was performed by omitting one study by turns. Subgroup analysis based on a priori hypothesis that is the analgesic effects of PVB and ESPB are related to surgical site. The Egger's test, as well as visual examination of the funnel plot were used to assess potential publication bias. RevMan (version 5.3; Cochrane Library, Oxford, UK) was used to perform meta-analyses, and STATA 14/MP (StataCorp., College Station, TX, USA) was used for conducting Egger's test (metabias module).

29. * Analysis of subgroups or subsets.

State any planned investigation of ‘subgroups’. Be clear and specific about which type of study or participant will be included in each group or covariate investigated. State the planned analytic approach.

Subgroup analysis by type of surgery (thoracic surgery and breast surgery) and predefined sources of heterogeneity was performed.

30. * Type and method of review.

Select the type of review, review method and health area from the lists below.

Type of review

Cost effectiveness
No

Diagnostic
No

Epidemiologic
No

Individual patient data (IPD) meta-analysis

No

Intervention
No

Living systematic review
No

Meta-analysis
Yes

Methodology
No

Narrative synthesis
No

Network meta-analysis
No

Pre-clinical
No

Prevention
No

Prognostic
No

Prospective meta-analysis (PMA)
No

Review of reviews
No

Service delivery
No

Synthesis of qualitative studies
No

Systematic review
Yes

Other
No

Health area of the review

Alcohol/substance misuse/abuse
No

Blood and immune system
No

Cancer
No

Cardiovascular
No

Care of the elderly
No

Child health
No

Complementary therapies
No

COVID-19
No

Crime and justice
No

Dental
No

Digestive system
No

Ear, nose and throat
No

Education
No

Endocrine and metabolic disorders
No

Eye disorders
No

General interest
No

Genetics
No

Health inequalities/health equity
No

Infections and infestations
No

International development
No

Mental health and behavioural conditions
No

Musculoskeletal
No

Neurological
No

Nursing
No

Obstetrics and gynaecology
No

Oral health
No

Palliative care

No

Perioperative care
Yes

Physiotherapy
No

Pregnancy and childbirth
No

Public health (including social determinants of health)
No

Rehabilitation
No

Respiratory disorders
No

Service delivery
No

Skin disorders
No

Social care
No

Surgery
No

Tropical Medicine
No

Urological
No

Wounds, injuries and accidents
No

Violence and abuse
No

31. Language.

Select each language individually to add it to the list below, use the bin icon to remove any added in error.
English

There is not an English language summary

32. * Country.

Select the country in which the review is being carried out. For multi-national collaborations select all the countries involved.

China

33. Other registration details.

Name any other organisation where the systematic review title or protocol is registered (e.g. Campbell, or The Joanna Briggs Institute) together with any unique identification number assigned by them. If extracted

data will be stored and made available through a repository such as the Systematic Review Data Repository (SRDR), details and a link should be included here. If none, leave blank.

34. Reference and/or URL for published protocol.

If the protocol for this review is published provide details (authors, title and journal details, preferably in Vancouver format)

Add web link to the published protocol.

Or, upload your published protocol here in pdf format. Note that the upload will be publicly accessible.

No I do not make this file publicly available until the review is complete

Please note that the information required in the PROSPERO registration form must be completed in full even if access to a protocol is given.

35. Dissemination plans.

Do you intend to publish the review on completion?

No

Give brief details of plans for communicating review findings.?

36. Keywords.

Give words or phrases that best describe the review. Separate keywords with a semicolon or new line. Keywords help PROSPERO users find your review (keywords do not appear in the public record but are included in searches). Be as specific and precise as possible. Avoid acronyms and abbreviations unless these are in wide use.

37. Details of any existing review of the same topic by the same authors.

If you are registering an update of an existing review give details of the earlier versions and include a full bibliographic reference, if available.

38. * Current review status.

Update review status when the review is completed and when it is published.New registrations must be ongoing so this field is not editable for initial submission.
Please provide anticipated publication date

Review_Ongoing

39. Any additional information.

Provide any other information relevant to the registration of this review.

?????????????????meta???

40. Details of final report/publication(s) or preprints if available.

Leave empty until publication details are available OR you have a link to a preprint (NOTE: this field is not editable for initial submission). List authors, title and journal details preferably in Vancouver format.

Give the link to the published review or preprint.
