# Supplementary material for: Postoperative analgesic effects of paravertebral block versus erector spinae plane block for thoracic and breast surgery: A meta-analysis
Source: PLoS One. 2021 Aug 25;16(8):e0256611. doi: 10.1371/journal.pone.0256611 (PMC8386864; doi:10.1371/journal.pone.0256611)
Supplement: S2 File — (DOC) [file pone.0256611.s005.doc]

**#1 Search**: ****((((erector spinae plane block[Title/Abstract]) OR (erector spinae[Title/Abstract])) OR (ESP[Title/Abstract])) OR (ESPB[Title/Abstract])) OR (ESP block[Title/Abstract])****

**#2 Search**: ****(((((paravertebral plane blocks[Title/Abstract]) OR (para-vertebral block[Title/Abstract])) OR (PVB[Title/Abstract])) ) OR (thoracic Paravertebral Block[Title/Abstract])) OR (TPVB[Title/Abstract])****

****#3 Search: (((((thoracic surgery[Title/Abstract]) OR (thoracoscopic surgery[Title/Abstract])) OR (thoracotomy[Title/Abstract])) OR (modified radical mastectomy[Title/Abstract])) OR (mastectomy[Title/Abstract])) OR (breast surgery[Title/Abstract])****

**#1 AND #2 AND **#3****
